# Clinical Benefit of Basal Insulin Analogue Treatment in Persons with Type 2 Diabetes Inadequately Controlled on Prior Insulin Therapy: A Prospective, Noninterventional, Multicenter Study

**DOI:** 10.1007/s13300-018-0378-4

**Published:** 2018-02-19

**Authors:** Jelica Bjekić-Macut, Teodora Beljić Živković, Radivoj Kocić

**Affiliations:** 1 Belgrade School of Medicine, Bezanijska Kosa University Medical Center, Division of Endocrinology, Diabetes and Metabolic disorders, University of Belgrade, Belgrade, Serbia; 2 Belgrade School of Medicine, “Zvezdara” University Medical Center, Division of Endocrinology, Diabetes and Metabolic disorders, University of Belgrade, Belgrade, Serbia; 3 Clinic for Endocrinology, Faculty of Medicine, University of Nis, Nis, Serbia

**Keywords:** Basal insulin analogue, Fasting plasma glucose, FPG, Glycated hemoglobin, HbA_1c_, Hypoglycemia, Insulin degludec, Insulin detemir, Insulin glargine, Type 2 diabetes

## Abstract

**Introduction:**

Basal insulin analogues offer persons with type 2 diabetes mellitus (T2DM) adequate glycemic control combined with a favorable safety profile. BASAL-BALI—a prospective, noninterventional, multicenter disease registry—assessed the effectiveness and safety of basal insulin analogues in adult Serbians with T2DM previously inadequately controlled on other insulin types.

**Methods:**

The primary objective was to assess the reduction in glycated hemoglobin (HbA_1c_) from basal insulin analogue initiation to the end of a 6-month observation period. Data collection was performed at three study visits: baseline, 3 months, and 6 months. All treatments and procedures were performed at the physicians’ discretion.

**Results:**

In total, 460 subjects were included. Mean diabetes duration was 11.6 ± 6.6 years. Late complications of diabetes were present in 67% of subjects and comorbidities in 85%. After 6 months, the mean reduction in HbA_1c_ was 1.8% (*p* < 0.01 vs. baseline); body weight (mean reduction of 0.9 kg, *p* < 0.01), waist circumference (1.5 cm, *p* < 0.01), and BMI (0.2 kg/m^2^, *p* < 0.01) were also reduced. A total of 49.1% of subjects reached their individualized HbA_1c_ treatment target, and 42.0% met the composite HbA_1c_ and fasting plasma glucose (FPG) target. The incidence of symptomatic hypoglycemia was reduced from 96.3% in the 6 months prior to initiating basal insulin analogues to 15.4% over the 6-month treatment period.

**Conclusion:**

Introducing basal insulin analogues in persons with T2DM previously inadequately controlled on other insulin types can significantly improve glycemic control and reduce the risk of hypoglycemia, without adversely affecting body weight.

**Funding:**

Sanofi, Serbia.

**Electronic supplementary material:**

The online version of this article (10.1007/s13300-018-0378-4) contains supplementary material, which is available to authorized users.

## Introduction

Diabetes is a major health issue in Europe, affecting 56.3 million adults in the broader European Region, with forecasts predicting this number to increase to almost 70 million by 2035 [1]. The prevalence of diabetes among adults in Serbia is 12.4%—substantially above the European average of 8.5% [1]. In those with type 2 diabetes mellitus (T2DM), lowering blood glucose may help to prevent vascular complications of the disease: the UK Prospective Diabetes Study showed that every percentage point decrease in glycated hemoglobin (HbA_1c_) reduced the risk of microvascular complications by 37%, and amputation or death from peripheral vascular disease by 43% [2]. The internationally recognized guidelines from the International Diabetes Federation (IDF), as well as the joint position statement from the American Association of Diabetes (ADA) and the European Association for the Study of Diabetes (EASD), recommend that HbA_1c_ levels should be kept below 7% (53.0 mmol/mol) [3, 4]. However, a lower target may be appropriate in some individuals, while a less stringent one should be set for those with comorbidities or other risk factors [3, 4].

In routine clinical practice, optimal glycemic control is rarely achieved, despite individualization of treatment goals. Insulin, while not generally considered first-line treatment in T2DM, should be seen as an essential therapy component in those not achieving their individualized HbA_1c_ target, despite intensive therapy with oral agents [4]. Initiating insulin treatment with basal insulin in persons with T2DM is recommended by international treatment guidelines from the IDF and the ADA/EASD [3, 4]. Human intermediate basal insulin (Neutral Protamine Hagedorn, NPH) is often the initial choice, but, with its duration of action of 12–18 h, it is associated with a risk of hypoglycemia [5] and may require multiple injections during the day. It also fails to mimic the physiological profile of insulin release and has broad intra- and inter-individual bioavailability [5].

Insulin analogues were developed to mimic the physiological profile of insulin secretion more closely, reduce hypoglycemia risk, and improve bioavailability [6]. Meta-analyses of clinical trials reported efficacy outcomes with basal insulin analogues to be mostly non-inferior or better than with other insulins, while the incidence of hypoglycemia (in particular nocturnal) was reduced [7, 8]. In the real-world setting, HbA_1c_ reductions associated with basal insulin analogues varied between studies within an approximate range of 0.3–1.5%, depending on prior treatment [9]. Few hypoglycemic episodes were reported [9]. Indeed, compared to NPH, basal insulin analogues have been shown to reduce the incidence of hypoglycemia (especially nocturnal) and weight gain—two main adverse effects that may be associated with intensifying diabetes treatment, and are a concern for both patients and physicians [6]. As fear of nocturnal hypoglycemia is also thought to be one of the major factors reducing adherence to treatment, those receiving basal insulin analogues may be more likely to adjust their insulin doses to achieve treatment goals [10].

The BASAL-BALI registry was a prospective, noninterventional disease registry that followed Serbian adults with T2DM initiated on basal insulin analogues after failing to achieve satisfactory glycemic control with human insulins. The aim of the study was to collect effectiveness and safety data relating to hypoglycemic episodes, and characterize the rationale for initiating treatment with basal insulin analogues.

## Methods

### Study Design and Population

BASAL-BALI was a prospective, noninterventional, multicenter study (conducted from April 2014 to September 2014) involving 45 physicians from 19 sites treating individuals with T2DM in Serbia. The participating sites were selected to ensure a regular geographic distribution across the country and representative settings of care, including both large university hospital centers and small local hospitals.

Forty-five endocrinologists/diabetologists were selected to participate, while general practitioners (GPs) were not included, since they are currently not authorized to prescribe insulin therapy in Serbia. The study included male or female T2DM individuals aged 18 years or more who had been on basal insulin analogues for 2–4 weeks at enrollment, were on stable doses of concomitant antidiabetic treatment, and who had previously been inadequately controlled on other types of insulins (HbA_1c_greater than 7% and persistent hypoglycemia with at least three documented episodes of plasma glucose values less than 3.5 mmol/L). To help eliminate bias, each physician was advised to include consecutive subjects who were suitable for the study.

### Ethics Approval

Signed informed consent was obtained from all participants. The study was approved by ethics committees and conducted in accordance with Good Clinical Practice and the Declaration of Helsinki. Women who were pregnant, breast-feeding, or planning to become pregnant during the study, persons with type 1 diabetes mellitus, and those already taking part in other studies were excluded. This noninterventional study did not interfere with everyday clinical practice and all treatments and procedures were performed at the physicians’ discretion.

### Objectives

The primary objective of the BASAL-BALI study was to assess the reduction in HbA_1c_ from the time of the basal insulin analogue initiation to the end of the 6-month observation period. Secondary objectives of the study included identifying the proportion of subjects who (1) reached their individualized HbA_1c_ target; (2) had a satisfactory response to treatment, defined as a decrease in HbA_1c_ of at least 0.5% or 1%; and (3) reached a HbA_1c_ level below 7% with no documented symptomatic hypoglycemia during the 6-month observation period. Other secondary objectives of this study were to (4) assess the rates of symptomatic, nocturnal, and severe hypoglycemia; (5) evaluate the reduction in fasting plasma glucose (FPG), as measured by self-monitoring of blood glucose (SMBG) and (6) the change in body weight; (7) collect information on the doses of basal insulin analogues used and the number of injections per day; (8) obtain data on the type and characteristics of concomitant oral antidiabetic (OAD) therapy and its influence on hypoglycemic event occurrence; (9) assess the effect of adherence to the individualized treatment regimen on treatment success (measured as the percentage of subjects achieving the glycemic goal of HbA_1c_ below 7% among those adherent or non-adherent to basal insulin dose adjustment); (10) assess the efficacy of basal insulin analogues in subjects with additional risk factors and comorbidity; (11) obtain the characteristics of subjects at the time of starting treatment with basal insulin analogues; and (12) describe the rationale behind the physician’s decision to initiate this therapy.

### Clinical Data Collected

Data collection was performed at each of the three study visits (i.e., at baseline, 3 months, and 6 months). At baseline it included evaluation of inclusion/exclusion criteria, socio-demographic data, diabetes history, complications and medication, concomitant disease and medication, body measurements (height, weight, waist circumference), vital signs (blood pressure), and laboratory data. Data on HbA_1c_ and FPG levels were collected retrospectively and were related to basal analogue initiation (i.e., 2–4 weeks prior to enrollment into the study). Investigators also described their rationale for basal insulin initiation, recorded individualized HbA_1c_ target and FPG values, and collected information on adverse events occurring during the 6-month period preceding initiation of basal insulin therapy (including type and frequency of hypoglycemic episodes, and duration and reasons for hospitalization).

At the 3- and 6-month visits, investigators collected data on body weight, waist circumference, blood pressure, glycemic status (HbA_1c_ and FPG), basal insulin analogue treatment (daily dose and number of injections), concomitant treatment (short-acting insulin and OADs), and the type and number of hypoglycemic episodes occurring since the previous visit. At the 6-month visit, which concluded the follow-up period, the investigators also collected data on hospitalizations and recorded their assessment of therapy, including whether HbA_1c_ and FPG targets had been reached, reasons for failure to reach these target (if applicable) and evaluation of SMBG levels.

### Statistical Analysis

All analyzed were performed on the basis of the intention-to-treat (ITT) principle. Depending of the type and number of examined parameters, the Chi-squared test, Mantel–Haenszel test, Student’s *t* test, Mann–Whitney test, Wilcoxon test, or ANOVA was used for data analysis. In all tests, an alpha level of 0.05 (*p* < 0.05) was considered statistically significant. Missing data were included in descriptive analysis; in other analyses, they were handled as missing data and excluded from the analysis. Characteristics of participants were included as covariates in the analyses, including age, gender, body mass index (BMI), central obesity, presence of late diabetes complications, hypoglycemia, duration of diabetes, and cardiovascular risk factors. Continuous data are presented as mean values with standard deviations (SD) and categorical data as absolute numbers (*n*) with percentages. Data analysis was performed using SPSS version 17 (Statistical Package for Social Sciences for Windows, SPSS Inc., Chicago, IL, USA).

## Results

**Fig. 1:**
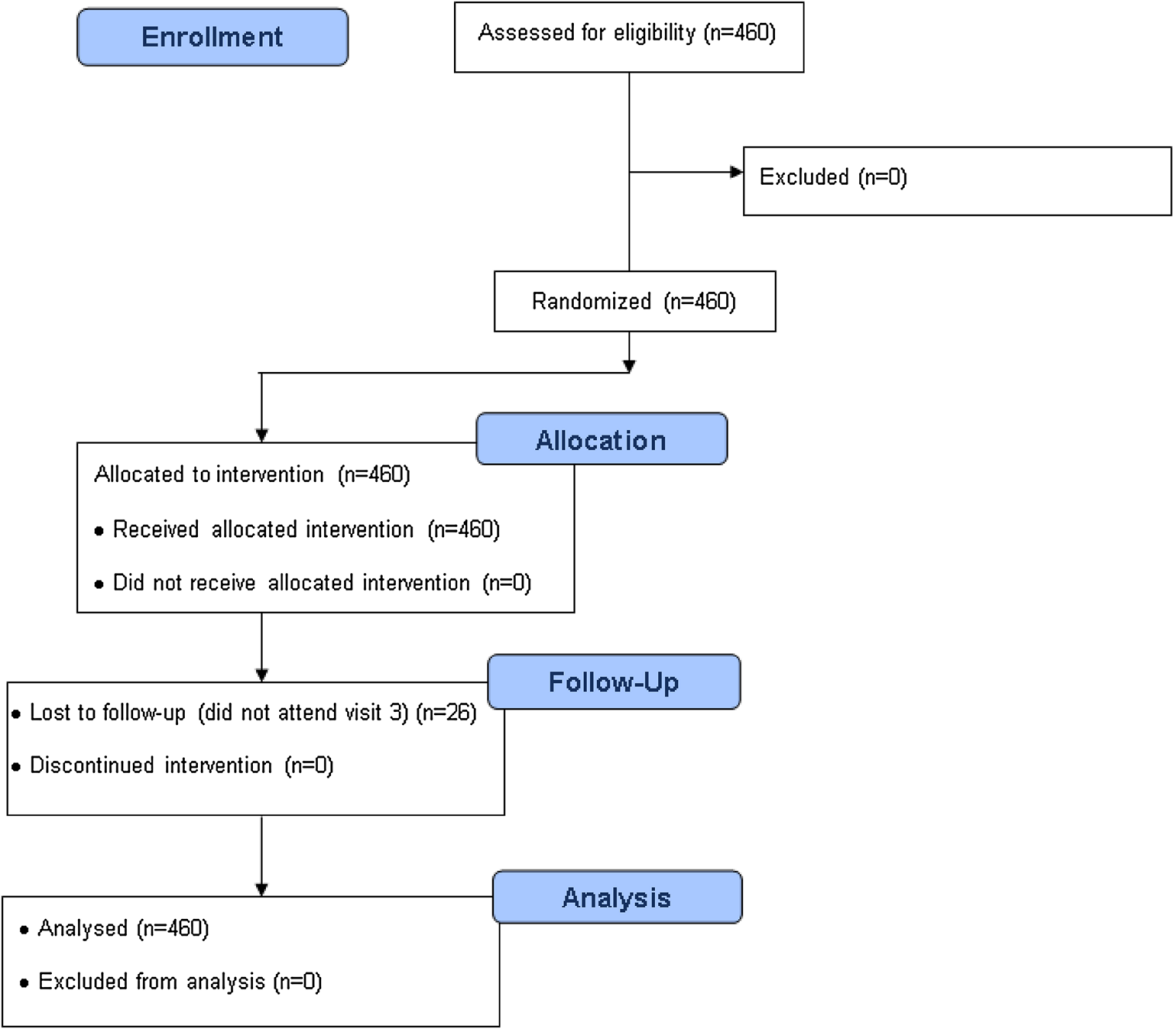
Participant flow in the BASAL-BALI study

A total of 460 subjects with T2DM who met the inclusion/exclusion criteria were included in the BASAL-BALI study, and 434 completed the 6-month follow-up. No violation of inclusion criteria or withdrawal of consent was reported, so all 460 subjects who entered the study were included in the analysis. The CONSORT diagram (Fig. 1) presents participant flow in the study.

### Baseline and Disease Characteristics in the Study Cohort

Most subjects were aged 55–65 years (mean age was 59.8 ± 10.2 years). Male subjects included in the study were significantly younger (58.26 ± 10.3 years), than female subjects (61.01 ± 10.0 years, *p* < 0.01). Abdominal obesity was present in 78.3% of subjects, based on ethnicity-specific waist circumference of at least 80 cm in women and at least 94 cm in men, as per 2006 IDF guidelines [11]. At enrollment, the mean duration of diabetes was 11.6 ± 6.6 years, with a median of 10.3 years.

Information on previous insulin therapy was collected for all study participants. Short-acting insulins had been used by 136 subjects (29.6%), intermediate- and long-acting insulins by 298 (64.8%), and premixes by 163 (35.4%). As short- and intermediate- or long-acting insulins may be combined in a single regimen to improve glycemic control, the total number of subjects previously treated with the three insulin types (597) exceeded the number of individuals enrolled in the study (460). Overall, mean duration of previous insulin therapy was 2.7 ± 3.3 years. Considering different insulin types, the average duration of therapy was 1.1 ± 2.5, 2.7 ± 3.3, and 1.5 ± 3.1 years for short-acting, long-acting insulins, and premix insulins, respectively. Daily doses of prior insulin therapy are available in the Supplementary Material, as are doses of basal insulin analogues administered while on study.

At baseline, mean systolic/diastolic blood pressure of study participants was 136.8 ± 15.8 mmHg/83.0 ± 9.1 mmHg. Chronic complications of diabetes mellitus were prevalent in this study cohort, with microvascular complications such as neuropathy (57.6%) and retinopathy (38.9%) being the most common. In addition, the majority of participants had significant comorbidities, including hypertension (72.1%) and dyslipidemia (72.6%).

Overall, 347 (75.4%) subjects received concomitant OADs, with biguanides being the most common (in 71.3%, 66.5%, and 65.6% of subjects at baseline, 3-month, and 6-month visits, respectively), followed by sulfonylureas (9.1%, 7.8%, and 7.6% of subjects), and other OADs (2.2%, 2.4%, and 2.4% of subjects). The type of OAD treatment did not significantly affect the incidence of hypoglycemic events (*p* = 0.23). Table 1 provides detailed characteristics of the study cohort at inclusion.

**Table 1:** Baseline characteristics of the study cohort

Characteristic	Value
Age (years), mean ± SD	59.8 ± 10.2
Gender, *n* (%)	
Male	189 (41.1%)
Female	271 (58.9%)
Duration of T2DM (years)	
Mean ± SD	11.6 ± 6.6
Median	10.3
Body mass index (kg/m^2^), mean ± SD	28.6 ± 4.7
Fasting plasma glucose (mmol/L), mean ± SD	9.8 ± 2.6
HbA_1c_ (%), mean ± SD	8.9 ± 1.3
Duration of previous insulin therapy (years), mean ± SD	2.7 ± 3.3
Type of previous insulin therapy, *n* (%)	
Short-acting	136 (29.6%)
Intermediate- and long-acting	298 (64.8%)
Premix	163 (35.4%)
Comorbidities, *n* (%)	
Hypertension	331 (72.1%)
Dyslipidemia	334 (72.6%)
Other	58 (12.6%)
Microvascular complications, *n* (%)	
Neuropathy	265 (57.6%)
Retinopathy	179 (38.9%)
Nephropathy	38 (8.3%)
Macrovascular complications, *n* (% of participants)	
Coronary artery disease	92 (20.0%)
Diabetic foot syndrome	24 (5.2%)
Other cardiovascular diseases	6 (1.3%)

### Change in HbA_1c_ Level During the Study Period (Primary Objective)

**Fig. 2:**
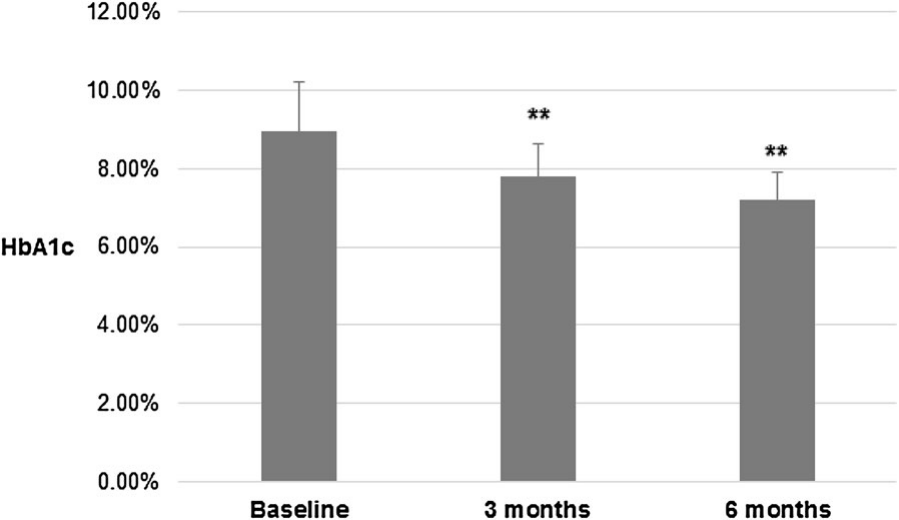
Reduction in glycated hemoglobin (HbA_1c_) levels following basal insulin initiation. Paired *t* test, **p < 0.01 vs previous visit

HbA_1c_ levels decreased significantly following basal insulin analogue initiation (Fig. 2), which was already evident at the 3-month visit (7.7 ± 0.7%, *p* < 0.01) compared with baseline (8.9 ± 1.3%), with a further reduction observed at 6 months (7.1 ± 0.7%, *p* < 0.01 vs. 3 months). Thus, the average reduction in HbA_1c_ observed during the 6-month treatment period was 1.8% (*p* < 0.01 vs. baseline).

### Secondary Objectives

**Fig. 3:**
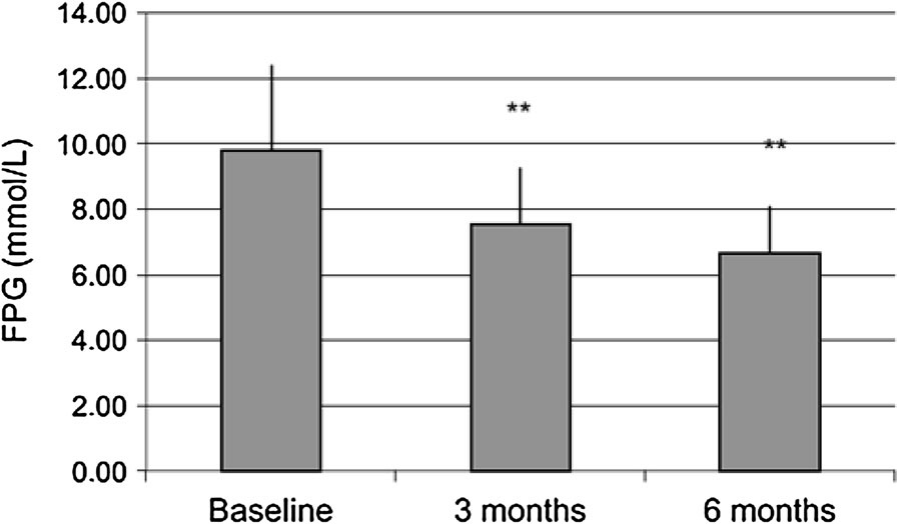
Reduction in mean fasting plasma glucose (FPG) levels following basal insulin initiation. Paired *t* test, **p < 0.01 vs previous visit

There was a significant reduction in FPG levels throughout the study duration (Fig. 3), with mean FPG levels reduced from 9.8 ± 2.6 mmol/L at the time of basal insulin analogue initiation to 7.5 ± 1.7 mmol/L at the 3-month visit (*p* < 0.01) and further to 6.7 ± 1.4 mmol/L at the 6-month visit (*p* < 0.01 vs. 3-month visit). At baseline, 25 subjects (5.4%) were already at the target FPG level. This number increased to 144 subjects (31.3%) at 3 months, and to 245 subjects (53.3%) at 6 months, the latter change being statistically significant (*p* < 0.01 vs. baseline and vs. the previous visit).

Body weight, waist circumference, and BMI were all significantly lower at the end of the study compared with the start of basal analogue treatment. Body weight was reduced from a mean of 82.4 ± 14.3 kg at baseline to 81.5 ± 13.5 kg at 6 months (*p* < 0.01). Over the same period, average waist circumference decreased from 94.9 ± 14.0 to 93.4 ± 13.5 cm (*p* < 0.01) and mean BMI decreased from 28.6 ± 4.7 to 28.4 ± 4.8 kg/m^2^ (*p* < 0.01).

The mean individualized HbA_1c_ target set for each subject by the physicians at study entry was 7.0 ± 0.4%. Most subjects (50.5%) had HbA_1c_ targets of 7.0% to below 7.5%, 31.3% had targets of 6.5% to below 7.0%, and 13.4% had targets of 7.5% to below 8.0%. At the 3-month visit, 53 (11.5%) subjects reached their individualized HbA_1c_ target, and this number increased significantly to 226 (49.1%, *p* < 0.01) at the 6-month visit. Although the individualized treatment targets were generally close to 7%, subjects who achieved a reduction in HbA_1c_ of at least 0.5% were considered as having a satisfactory response to treatment and there was a significant increase in the proportion of these subjects, from 61.3% at 3 months to 81.3% at 6 months (*p* < 0.01). Furthermore, a substantial proportion of subjects achieved decreases in HbA_1c_ of more than 1.0%—again, significantly more subjects did so at 6 months (67.2%) than at 3 months (40.4%, *p* < 0.01).

**Fig. 4:**
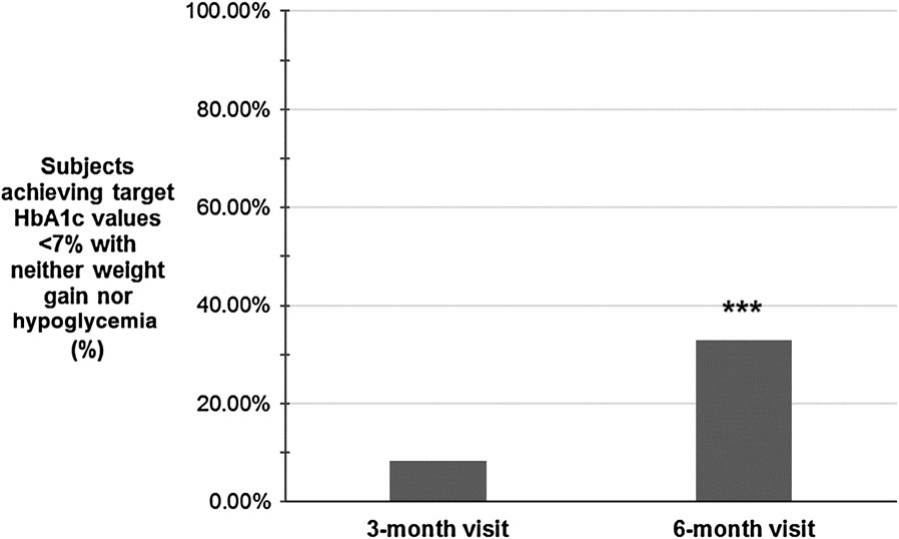
Proportion of subjects who achieved HbA_1c_ target values below 7% without experiencing hypoglycemia or weight gain. Data were available for 431 participants (93.7%) at the 3-month visit and for 425 (92.4%) at the 6-month visit. Wilcoxon signed ranks test, **p < 0.001 vs previous visit

When both HbA_1c_ and FPG (mean target of 6.5 ± 0.7 mmol/L with a median of 6.5 mmol/L) were considered as a composite target, the proportion of subjects achieving individualized treatment goals also increased significantly from the 3-month (8.3%) to the 6-month visit (42.0%, *p* < 0.01). Another composite measure of interest was the proportion of individuals achieving target HbA_1c_ values below 7% without experiencing hypoglycemia or weight gain (Fig. 4). Among individuals with available data, this increased significantly from the 3-month (*n* = 36, 8.4%) to the 6-month visit (*n* = 140, 32.9%, *p* < 0.001).

### Factors Affecting Subjects’ Ability to Reach HbA_1c_ Targets

Unsurprisingly, adherence to treatment played a substantial part in achieving HbA_1c_ targets. Among subjects reaching HbA_1c_ below 7.0%, significantly more followed the physician’s instructions on insulin titration (*n* = 89, 19.3%) compared with subjects who did not titrate insulin as instructed (*n* = 77, 16.7%, *p* < 0.05).

Subjects with late complications of diabetes (*n* = 310, 67.4%) appeared less likely to reach HbA_1c_ levels below 7% than those without such complications—only 114 (39.7%) of subjects with late complications achieved HbA_1c_ below 7% compared with 75 (54.3%) of those without such complications (*p* < 0.01). However, when individualized HbA_1c_ targets were evaluated, there was no significant difference in the proportion of subjects reaching their targets according to the presence of late complications (57.9% without late complications of diabetes vs. 50.8% with late complications, *p* = 0.17).

The presence of comorbidities, affecting 392 (85.2%) subjects, had a similar adverse effect on subjects’ ability to achieve lower HbA_1c_ levels—the proportion of subjects with no comorbidities reaching HbA_1c_ below 7.0% (*n* = 42, 66.7%) was significantly higher than the proportion of those with comorbidities who reached this target (*n* = 147, 40.6%, *p* < 0.01). The presence of comorbidities had a similar negative impact on reaching individualized HbA_1c_ targets at the end of the follow-up. At the 6-month visit significantly more subjects without comorbidities reached their individual HbA_1c_ target (68.2%) compared with subjects who had comorbidities (50.5%, *p* < 0.01).

### Safety of Basal Insulin Analogues

During the 6 months prior to initiating basal insulin analogues, 443 subjects (96.3%) reported hypoglycemic events (Table 2). After 6 months of basal insulin analogue treatment, the incidence of symptomatic hypoglycemia was substantially lower, affecting 71 subjects (15.4%; *p* < 0.001 vs. 6 months before insulin analogue treatment). Similarly, the incidence of serious hypoglycemic episodes and nocturnal hypoglycemia was lower during the 6-month study period than over the same time period preceding basal insulin analogue treatment (*p* < 0.001 vs. 6 months before insulin analogue treatment).

**Table 2:** Change in hypoglycemia incidence and rate following basal insulin analogue treatment (6 months before treatment and 6 months after the start of treatment)

	Documented symptomatic hypoglycemia		Serious hypoglycemia		Nocturnal hypoglycemia	
	6 months before	6 months after	6 months before	6 months after	6 months before	6 months after
Incidence of hypoglycemia; *n* (% of subjects with ≥ 1 symptomatic episode)	443 (93.9%)	71 (15.4%)**	144 (31.4%)	1 (0.2%)**	217 (47.3%)	18 (3.9%)**
Number of events per patient-year	14.5	1.56**	2.29	0.004**	3.34	0.25**

Data are presented as *n*/% or number of events

Wilcoxon signed ranks test; **p < 0.001 vs. 6 months before treatment

## Discussion

Results from the BASAL-BALI study showed that switching persons with T2DM who were inadequately controlled on human insulins to basal insulin analogues was associated with significant improvement in glycemic control, measured in terms of reduction in HbA_1c_ and FPG levels. Late complications of diabetes were present in 67.4% of subjects and comorbidities in 85.2%. Reduction in HbA_1c_ was more efficient in subjects with no complications. The improvement in glycemic control was achieved without an adverse impact on body weight. Importantly, the incidence of hypoglycemic episodes during 6 months of treatment with basal insulin analogues was substantially reduced compared with the preceding 6-month period, when subjects received other insulin types.

In the BASAL-BALI study, mean HbA_1c_ levels were reduced by 1.8% over 6 months after switching to basal insulin analogues. Prior research suggests that this reduction in HbA_1c_ may have long-term benefits: an analysis of the UK Prospective Diabetes Study showed that a 1% reduction in HbA_1c_ was linked to a 14% reduction in the risk of myocardial infarction, a 21% reduction in mortality related to diabetes, and a 43% decrease in the risk of amputation or death from peripheral vascular disease [2]. Furthermore, compared with the 6-month period prior to basal insulin analogue initiation, the incidence of hypoglycemic episodes was lower during treatment. Hypoglycemic events during the 6 months before basal insulin analogue introduction were reported in 443 (96.3%) subjects, while the number of affected subjects decreased to 56 (12.8%) at the 3-month visit and 53 (11.5%) at the 6-month visit. As hypoglycemia often represents a barrier to optimization of diabetes therapy, this result means that persons with T2DM receiving basal insulin analogues may be able to make better use of the benefits of insulin-based treatment.

Our results are well aligned with the expectations of physicians, who aimed to achieve good glycemic control without hypoglycemia or weight gain when initiating persons with T2DM on basal insulin analogues. Indeed, an early switch to basal insulin analogues may be recommended, in line with recent studies suggesting that early initiation of this therapy may provide sustained long-term glycemic control, that is improved compared with standard of care, thus offering persons with T2DM more effective protection from the toxic effects of hyperglycemia [12].

The reduction in HbA_1c_ observed in this study is within the broad range reported with basal insulin analogues in other real-world studies. ORBIT—a multicenter, prospective, 6-month registry-based study conducted in China—showed that persons with T2DM previously inadequately controlled on OADs, following initiation of basal insulin analogues, experienced a mean reduction in HbA_1c_ of 2%, with 48% of participants achieving treatment targets [13]. In insulin-naïve subjects included in the Swedish National Diabetes Register, the mean decrease in HbA_1c_ over 12 months of treatment with basal insulin analogues was 7 ± 17 mmol/mol [14] (0.6 ± 1.5% [15]) for insulin glargine and 7 ± 18 mmol/mol [14] (0.6 ± 1.6% [15]) for insulin detemir. In a UK-based observational study of insulin-naïve subjects utilizing the Health Improvement Network medical records database, the mean reduction in HbA_1c_ was 1.2 ± 1.7% over 12 months of treatment with insulin glargine and 1.0 ± 2.0% in those treated with insulin detemir [16]. Thus, the reduction in HbA_1c_ in our study was more pronounced than in the UK study, and much higher than in the Swedish one, although the Swedish cohort had the lowest baseline HbA_1c_ levels among the three. The doses of insulin glargine, the most commonly used basal insulin analogue in our study (*n* = 434, 94.3% at baseline), was similar (3100.314 ± 110.1298 U/kg body weight/day at baseline) to that in the Swedish study (0.33 ± 0.16 U/kg body weight/day) [14], but substantially lower than that in the UK one (0.56 ± 0.40 U/kg body weight/day) [16]. Thus, our results, showing meaningful improvement in glucoregulation in a population already on insulin treatment, should be taken into relevant consideration.

The number of participants achieving treatment targets is difficult to compare across studies because of differences in target definitions. For example, in the aforementioned UK study by Gordon et al., only about a third of participants treated with basal insulin analogues achieved HbA_1c_ values no greater than 7.5% (30% in the glargine group and 28% in the detemir group) [16]—substantially less than the 49.1% reaching individualized HbA_1c_ target in our study, even though the average target was lower than the 7.5% used in the UK study. Our results were also more favorable in terms of the proportion of subjects with satisfactory response to treatment: 67.2% of subjects achieved HbA_1c_ reduction of at least 1%, compared with 57% of glargine-treated and 39% of detemir-treated subjects in the UK study [16]. It is, however, worth noting that we present 6-month outcomes, while Gordon et al. reported 12-month outcomes [16].

The substantial proportion of participants (42%) reaching the composite HbA_1c_ and FPG endpoint target indicates that basal analogue treatment with concomitant OADs could be a therapeutic option to consider after human insulin options. This subgroup of persons with T2DM, in whom the HbA_1c_ target was not reached despite satisfactory FPG, could be candidates for further therapy intensification with prandial insulins or some newer therapeutic options.

The safety profile of basal insulin analogues emerging from the BASAL-BALI study appears more favorable than that of other insulin types, based on the substantially lower rate of hypoglycemic episodes during treatment, than during the 6 months prior to treatment initiation. In the clinical trial setting, the use of these analogues has also been associated with fewer hypoglycemic episodes compared with NPH insulin, especially when comparing nocturnal hypoglycemia rates [17, 18]. In our study, good glycemic control was achieved with no hypoglycemic episodes in nearly a fifth of participants. Similarly, a meta-analysis of trials comparing insulin glargine with NPH showed that the incidence of nocturnal hypoglycemia in subjects reaching HbA_1c _of 7% or below was significantly lower among those treated with glargine [17].

The type of concomitant OAD treatment (biguanide or sulfonylurea) did not significantly affect the rate of hypoglycemic events in our study; however, a pooled analysis from 15 treat-to-target randomized controlled trials, where participants received insulin glargine, and metformin, a sulfonylurea, or both, showed that the rates of overall and nocturnal hypoglycemia with insulin glargine plus metformin were generally lowest, while the highest rates were observed in participants treated with insulin glargine combined with both metformin and a sulfonylurea [19].

A small but significant decrease in body weight was seen in our study, despite improved glycemic control following insulin initiation usually being linked to weight gain. Indeed, increases in body weight or BMI with basal insulin have been observed in other real-world studies [13, 14, 16]. The weight gain was generally less prominent with basal insulin analogues than with NPH [13, 14, 16], although the difference was only statistically significant in some studies [13]. Thus, the average weight reduction observed in our study, although small, is more pronounced than in other studies. The decrease in body weight could have several explanations, including subjects’ adherence to physicians’ recommendations on dose titration and dietary measures, the high proportion of subjects concomitantly taking biguanides, and the low incidence of hypoglycemia that could reduce snacking.

Our study has several limitations. First, as only participants with available HbA_1c_ at the time of basal insulin analogue initiation were included in the study, we cannot be certain if subject selection was completely free of bias. Second, HbA_1c_ was not measured centrally and the quantification method was not specified, so that differences in laboratory methods between institutions cannot be ruled out. Third, the study was based on participants’ medical records, recorded by the physicians, leading to a substantial amount of missing data for a number of variables, including basic ones such as gender and body weight. In particular, data on hypoglycemia relied on the subject diaries, possibly leading to under-reporting of hypoglycemic episodes. Furthermore, the total number of study participants was 460, which is a sample size smaller than in some other similar studies in this field. Finally, we followed subjects for only 6 months—a relatively short time, considering diabetes is a chronic condition.

Despite the aforementioned limitations, the BASAL-BALI study provided valuable information on the real-world effectiveness and safety of basal insulin analogues in persons with T2DM previously inadequately controlled on other insulin regimens. The results presented suggest that introducing basal insulin analogues in these persons can significantly improve glycemic control and reduce the risk of hypoglycemia, with no deleterious impact on body weight. Satisfactory responses to treatment can be achieved in a large proportion of persons with T2DM, with the vast majority achieving at least a 1% reduction in HbA_1c_ levels. Thus, our data suggest that the main physician expectations with regard to switching persons with T2DM to basal insulin analogues—achieving good glycemic control without hypoglycemia or weight gain—can indeed be met in routine clinical practice, outside the clinical trial setting.

## Conclusions

The BASAL-BALI study provided real-world effectiveness and safety data suggesting that basal insulin analogues may be the preferred treatment option for persons with T2DM who do not achieve good glycemic control with human insulins. Future studies of factors that affect adherence to insulin treatment, and initiatives aiming to improve it, could allow more persons with T2DM to achieve optimal glycemic control and thus fully benefit from treatment with basal insulin analogues.

## Electronic supplementary material

Below is the link to the electronic supplementary material.

Supplementary material 1 (PDF 66 kb)
