# Author Correction: Clinical Benefit of Basal Insulin Analogue Treatment in Persons with Type 2 Diabetes Inadequately Controlled on Prior Insulin Therapy: A Prospective, Noninterventional, Multicenter Study

**DOI:** 10.1007/s13300-018-0406-4

**Published:** 2018-03-22

**Authors:** Jelica Bjekić-Macut, Teodora Beljić Živković, Radivoj Kocić

**Affiliations:** 1 Belgrade School of Medicine, Bezanijska Kosa University Medical Center, Division of Endocrinology, Diabetes and Metabolic disorders, University of Belgrade, Belgrade, Serbia; 2 Belgrade School of Medicine, “Zvezdara” University Medical Center, Division of Endocrinology, Diabetes and Metabolic disorders, University of Belgrade, Belgrade, Serbia; 3 Clinic for Endocrinology, Faculty of Medicine, University of Nis, Nis, Serbia

## Author Correction to: Diabetes Ther 10.1007/s13300-018-0378-4

In the original publication, values of the doses of insulin glargine, the most commonly used basal insulin analogue under the ‘Discussion’ section was incorrectly published.

The sentence “…was similar (3100.314 ± 110.1298 U/kg body weight/day at baseline) to that in the Swedish study (0.33 ± 0.16 U/kg body weight/day)”.

Should actually say “…was similar (0.31 ± 0.12 U/kg body weight/day at baseline) to that in the Swedish study (0.33 ± 0.16 U/kg body weight/day)”.

