# Supplementary material for: Clinical Benefit of Basal Insulin Analogue Treatment in Persons with Type 2 Diabetes Inadequately Controlled on Prior Insulin Therapy: A Prospective, Noninterventional, Multicenter Study
Source: Diabetes Ther. 2018 Feb 19;9(2):651–62. doi: 10.1007/s13300-018-0378-4 (PMC6104277; doi:10.1007/s13300-018-0378-4)
Supplement: Supplementary file 1 — Supplementary material 1 (PDF 66 kb) [file 13300_2018_378_MOESM1_ESM.pdf]

Supplementary Material to '**Clinical Benefit of Basal Insulin Analogue Treatment in Persons with Type 2 Diabetes Inadequately Controlled on prior insulin therapy: A Prospective, Non-interventional, Multicentre Study**' by Jelica Bjekic-Macut, Teodora Beljić Živković and Radivoj Kocic

Insulin therapy prior to study entry

Table 1. Type and average daily dose of short-acting insulins

	N	%	Number of daily doses	Total daily dose (U)
Not used	324	70.4	-	-
Not defined	65	14.1	-	-
Actrapid®	51	11.1	2.8 ± 0.5 (3)	27.4 ± 14.4
Apidra®	1	0.2	-	-
Humalog®	2	0.4	2.5 ± 0.7 (3)	36.0 ± 31.0
Insuman Rapid®	9	2.0	2.6 ± 0.7 (3)	22.6 ± 15.0
Novo Rapid®	8	1.7	2.67 ± 0.6 (3)	31.3 ± 15.0
Total	460	100.0	-	-

Data are presented as mean ± SD (median)

Table 2. Type and average daily dose of intermedium- and long-acting insulins

	N	%	Number of daily doses	Total daily dose (U)
Not used	162	35.2	-	-
Not defined	119	25.9	-	-
Humulin NPH®	2	0.4	1.2 ± 0.5 (1)	37.5 ± 9.1
Insulatard®	145	31.5	1.2 ± 0.5 (1)	29.7 ± 11.8
Insuman Basal®	32	7.0	1.29 ± 0.5 (1)	31.7 ± 10.8
Total	460	100.0	-	-

Data are presented as mean ± SD (median)

Table 3. Type and average daily dose of premixed insulins

	N	%	Number of daily doses	Total daily dose (U)
Not used	297	64.6	-	-
Not defined	66	14.3		
Humalog® Mix 50	2	0.4	2.0 ± 1.4 (2)	32.5 ± 30.0
Humalog® Mix 25	0	0.0	-	-
Humulin® M3	4	0.9	1.95 ± 0.5 (2)	40.5 ± 7.1
Insuman® Comb 25	26	5.7	1.85 ± 0.3 (2)	41.5 ± 11.7
Mixtard® 30	44	9.6	1.80 ± 0.5 (2)	46.1 ± 14.8
Novo Mix® 30	21	4.6	2.2 ± 0.4 (2)	47.1 ± 11.7
Total	460	100.0	-	-

Data are shown as mean ± SD (median)

Doses of basal insulin analogues while on study

Type and daily doses of basal insulin analogues used at each study visit are shown in Table 3. Lantus® was the most common basal insulin analogue used at baseline (n=434, 94.3%). The average daily doses did not significantly differ between basal analogues. The total daily dose of basal analogues significantly increased at each consecutive visit (p<0.01)

Table 4. Doses of basal insulin analogues used per day

	Baseline		3-month visit		6-month visit	
	N (%)	Total daily dose (U); mean ± SD	N (%)	Total daily dose (U); mean ± SD	N (%)	Total daily dose (U); mean ± SD
Missing	3 (0.7)	-	25 (5.4)	-	23 (5.0)	-
Lantus®	434 (94.3)	31.4 ± 11.98	414 (90.0)	32.7 ± 11.6	418 (90.8)	34.5 ± 12.4
Levemir®	23 (5.0)	29.1 ± 8.8	21 (4.6)	31.2 ± 10.7	19 (4.2)	33.3 ± 11.2
Total	460 (100.0)	29.7 ± 11.3	460 (100.0)	31.6 ± 11.6**	460 (100.0)	33.8 ± 11.6**

Student's t-test: NS between basal analogues at each visit, **p<0.01 vs previous visit

Type of analogue and the number of daily injections reported at each study visit are shown in Table 5. There was no significant difference in the number of daily doses between consecutive study visits. Twice-daily administration was significantly more common in the Levemir® than the Lantus® group at all three study visits (p<0.05).

Table 5. Type of basal analogue and the number of daily doses

	Baseline		3-month visit		6-month visit	
	1	2	1	2	1	2
Lantus®	98.9%	1.1%*	98.5%	1.5%*	98.5%	1.5%*
Levemir®	86.7%	13.3%	84.6%	15.4%	83.3%	16.7%
Total	98.3%	1.7%	97.8%	2.2%	97.8%	2.2%

NS between visits, *p<0.05 vs. Levemir

When analysing total daily dose of insulin (basal + prandial), the Basal Bali study showed that after switching to a basal insulin analogue the total daily dose of insulin was significantly lower (43.7 ± 19.6 U/day vs. 38.9 ± 18.8 U/day; p<0.01). However, due to the increase in the dose of basal analogues over the study period (see Table 4), there was no significant difference in total daily insulin doses after 6 months (44.1 ± 20.8 U/day) compared to baseline.
